# What Do Football Players Look at? An Eye-Tracking Analysis of the Visual Fixations of Players in 11 v 11 Elite Football Match Play

**DOI:** 10.3389/fpsyg.2020.562995

**Published:** 2020-10-16

**Authors:** Karl Marius Aksum, Lukas Magnaguagno, Christian Thue Bjørndal, Geir Jordet

**Affiliations:** ^1^ Institute of Sport and Social Sciences, Norwegian School of Sport Sciences, Oslo, Norway; ^2^ Institute of Sport Science, University of Bern, Bern, Switzerland

**Keywords:** football (soccer), perception, exploratory, ecological, experts, *in situ*, visual fixations

## Abstract

Current knowledge of gaze behavior in football has primarily originated from eye-tracking research in laboratory settings. Using eye-tracking with elite players in a real-world 11 v 11 football game, this exploratory case study examined the visual fixations of midfield players in the Norwegian premier league. A total of 2,832 fixations by five players, aged 17–23 years (*M* = 19.84), were analyzed. Our results show that elite football midfielders increased their fixation duration when more information sources became available to them. Additionally, participants used shorter fixation durations than previously reported in laboratory studies. Furthermore, significant differences in gaze behavior between the attack and defense phases were found for both areas of interest and fixation location. Lastly, fixation locations were mainly on the ball, opponent, and teammate category and the player in possession of the ball. Combined, the results of this study enhance the knowledge of how elite footballers use their vision when playing under actual match-play conditions. They also suggest that laboratory designs may not be able to capture the dynamic environment that footballers experience in competition.

## Introduction

Visual perception in sport has attracted widespread interest from researchers and practitioners alike (McGuckian et al., 2018b). Research has generally shown that expert athletes have superior perceptual skills compared to non-experts (Mann et al., 2007). Specifically, expert athletes engage in more effective visual search strategies and focus on more relevant areas compared to less skilled athletes (Williams et al., 1999). This behavior has been replicated in a wide variety of sports and tasks, including football (Savelsbergh et al., 2002), tennis (Murray and Hunfalvay, 2017), handball (Rivilla-García et al., 2013), and volleyball (Piras et al., 2014). Expert athletes have also been shown to be more accurate in decision-making and faster in anticipating future events compared to less skilled athletes (Mann et al., 2007).

A fundamental prerequisite for visual perception is gaze behavior, which is thought to “optimize visual information processing which allows an optimal coupling between perception and action” (Klostermann and Moeinirad, 2020, p. 146). Gaze behavior research commonly distinguishes between smooth pursuits, saccades, pursuit tracking, and fixations (Duchowski, 2007). Smooth pursuits describe the slow-movement tracking of an object; for example, following your finger as it slowly passes by your head while keeping your head still (Williams et al., 1999). A saccade is a very rapid, twitchy movement of the eyes from one position to another and can be understood as a transition between fixations (Schmidt and Lee, 2005). Although previous research has proposed that no information intake occurs during saccades (Duchowski, 2007), more recent research suggests that vision is clear and stable during saccadic eye movements (Binda and Morrone, 2018).

Fixations are especially central to understanding the gaze behavior that underpins sports performance. Fixations are eye movements that stabilize the retina over a stationary object and have been described as “pauses over informative regions of interest” (Salvucci and Goldberg, 2000, p. 71). The duration and location of these fixations vary depending on the type of sport and are used to extract relevant information for decision-making and action (Hüttermann et al., 2018). The ability to apply gaze fixations correctly is, therefore, highly relevant in dynamic team sports, such as football, where anticipation is integral to athletes’ playing ability and skill level (Hüttermann et al., 2018).

An extensive number of studies reporting on gaze behavior in football have focused on the number, duration, and location of fixations in different video-simulated football scenarios conducted in laboratory settings (McGuckian et al., 2018b). Much of this research has attempted to investigate differences in gaze behavior between football players at different levels and experience. In their recent review of expertise-related differences in gaze behavior in sport, Klostermann and Moeinirad (2020) showed that empirical evidence on expertise-related differences in gaze behavior has declined in recent years with heterogeneous findings related to fixation duration and the number of fixations. The most prevalent finding relates to differences in gaze location and quiet eye duration (relevant for less dynamic sports or tasks), which was found to be longer for experts than intermediates and novices (Klostermann and Moeinirad, 2020).

Only a few studies have attempted to understand how visual fixations in football vary as a consequence of different task constraints (i.e., distance to the ball, attack v defense, number of players, and viewing perspective). For example, studies of simulated 11 v 11 play have shown that the number of fixations increases, the duration of fixations decreases, and the location of fixations is directed toward more objects of information when the ball is far from the player (Roca et al., 2013; Vater et al., 2016). When the ball travels closer, the location of gaze becomes more centrally focused toward the player in possession (PiP; Roca et al., 2013). Similarly, when players experience increased time constraints, they tend to focus their gaze centrally while using their peripheral vision to extract information from the positioning and movements of other players (Vaeyens et al., 2007). This type of gaze behavior is called a “foveal spot” (Vater et al., 2019). The main advantage of this type of gaze strategy is that information is processed faster peripherally, meaning that relying on peripheral vision in time-constrained situations might be advantageous (Vaeyens et al., 2007).

The representativeness of the experimental tasks has also been shown to influence the gaze behavior of athletes at different levels, with increased representativeness mediating expertise effects in gaze location (Klostermann and Moeinirad, 2020). For example, Mann et al. (2009) showed how viewing perspective influenced the gaze strategies of football players, where players spent more time observing open space and had more fixations of shorter duration from an aerial perspective than a playing perspective. The extent to which it is possible to transfer findings on gaze behavior in experimentally controlled situations to gaze behavior in real sports competitions remains unclear (Hüttermann et al., 2018). A reason for the limited representativeness is because it is difficult for experimental tasks conducted in laboratory settings to account fully for the dynamic performance context experienced by athletes (Pinder et al., 2011).

In football, actions are based on a complex array of visual, auditory, kinesthetic, and somatic senses (Headrick et al., 2015); particular task constraints, such as defensive pressure from opponents and position on the field (Pinder et al., 2015); and environmental constraints, such as different playing surfaces and weather conditions (Renshaw et al., 2019). Furthermore, in field-based studies, gaze behavior is not examined in isolation from the flow of motor movement or behavior, which may help develop knowledge about the coupling of perception and action (Hüttermann et al., 2018). There has, therefore, been a call by researchers to study gaze behaviors in environments representative of the specific performance context (Dicks et al., 2010; Eldridge et al., 2013; Klostermann and Moeinirad, 2020). Consequently, *in situ* designs using eye-trackers in mini-states of the respective sports have been conducted in basketball (van Maarseveen et al., 2017), ice hockey (Martell and Vickers, 2004), and futsal (Corrêa et al., 2020).

In an attempt to bridge this gap in football research, a recent study had 20 team sport athletes and 20 individual sport athletes perform a football-specific decision-making task using a motor response in front of an immersive screen (Hüttermann et al., 2019). Surprisingly, although the football players made more correct pass decisions, they did not show better attentional and perceptual performance compared to the participants from other team sports (Hüttermann et al., 2019). Although the study attempted to design an experimental task more representative of football performance, they used pictures of players on the screen and, in doing so, arguably failed to capture the dynamics inherent to the actual performance context.

Most studies conducted during real-world football match play have limited their focus to visual exploratory behaviors, such as the frequency of head movements (scanning) directed away from the ball (e.g., Jordet, 2005; Eldridge et al., 2013; Jordet et al., 2013; McGuckian et al., 2018a, 2020), neglecting the actual gaze behavior properties of football players, or restricting gaze behavior research to set plays (Klostermann and Moeinirad, 2020). In one of the few examples of field-based studies of gaze behavior in football, Nagano et al. (2004) reported the fixations of four experts and four novices in a 1 v 1 defense situation. They found that expert players conducted systematic visual search behaviors in which they fixated less exclusively on the ball compared to novice players. Nevertheless, the simulated game situation (1 v 1) was dissimilar to the spatial, temporal, mental, and physical demands that football players face during real competitive situations. To our knowledge, no study has examined the gaze behaviors of football players outside of standardized situations, more specifically during competitive 11 v 11 match play.

Based on this gap in the literature, this exploratory case study aimed to expand our understanding of the specific gaze behaviors of football players in a representative performance context. More specifically, we investigated the duration, location, and context of visual fixations of five elite-level football players in 11 v 11 match play. We collected data on gaze behaviors using modern eye-tracking technology in a real-world football context. The use of eye-tracking may provide a powerful balance between ecological validity and experimental control (Klostermann and Moeinirad, 2020). Furthermore, studying the gaze behavior of skilled athletes could provide unique insights into the underlying processes of complex movement behavior and provide valuable guidelines for practitioners, as well as a basis for further studies (Klostermann and Moeinirad, 2020).

## Materials and Methods

### Participants

Five male football players, aged 17–23 years (*M* = 19.84, *SD* = 2.52), from two Norwegian premier league clubs, consented to participate in the study. All players were part of the first-team squad of their respective team, and all had played for Norway’s under-21 national team, suggesting that they were regarded as being among the most talented players in their age group.^1^ Participants were chosen based on their playing position as central midfielders. Players in this position are often surrounded by both teammates and opponents in every direction and are the most central players in attacking build-up play (Clemente et al., 2015), forcing these players to explore their surroundings constantly for optimal performance. The experiment was approved by the Norwegian Centre for Research Data (NSD), reference number 52593. All participants signed a written informed consent form in accordance with the General Data Protection Regulation and the Declaration of Helsinki prior to data collection.

### Procedure

Data were gathered during the competitive season. The two teams that participated in the study were contacted by email and telephone. Subsequent meetings with the coaching staff of both teams were conducted by the first and fourth authors, and a date for the two separate data collection sessions was agreed upon. Two pilot studies on elite youth players were conducted in the weeks leading up to the first data collection session. These pilot studies revealed the importance of having somewhat similar lighting and weather throughout the entire data collection process. Fortunately, the agreed-upon dates featured favorable weather conditions, so that sunlight and rain did not negatively impact eye-movement detection.

Data were gathered during two training matches of 11 v 11 match play on a full-size pitch. Both matches were played on the training pitches of the two respective clubs. One of the teams played against a local third division team, while the other team played an internal training match consisting of players from the first-team squad. The matches were played with standard association football rules, and there were no coach interventions in either of the matches after the matches had started.

All players familiarized themselves with and tested the equipment prior to the warm-up. Before the data collection started, the participants donned eye-tracking units, so they could be fitted and calibrated individually. This process took about 3 min. Three players were recorded for 20 min, and two players were recorded for 10 min. The difference in the duration was due to (a) the duration of the match, (b) the duration of the fitting process, and (c) the battery from the eye tracker became detached from one of the players during play and had to be reattached and recalibrated. Because this study did not analyze individual differences, we decided to include all recorded data irrespective of duration.

### Equipment

**Figure 1:**
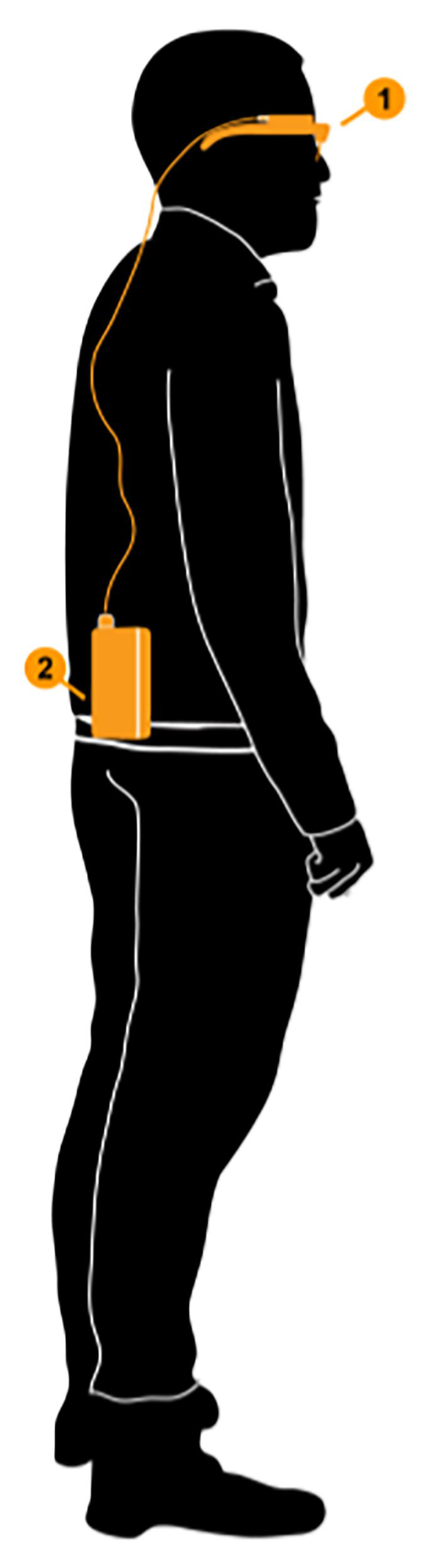
The head unit (1) and recording unit (2) of the Tobii Pro Glasses 2. Printed with permission.

A Tobii Pro Glasses 2-eye tracker was used to assess the players’ gaze behavior. The device consists of a head unit and a recording unit (see Figure 1). The camera on the head unit had a resolution of 1,920 × 1,080 at 25 frames per second. The recording unit was attached either on the player’s shorts or upper back. This enabled the participants to move freely.

The eye-tracking device used in this study used gaze-overlaid video, meaning that the device recorded wherever the participant’s point of gaze was fixed within the video display (Holmqvist et al., 2011). Thereafter, we used a fixation filter to look at all the fixations performed by the players. The Tobii Fixation Filter is a velocity-based algorithm for fixation detection in data as slow as 30 and 50 Hz. These velocity algorithms typically include smooth pursuits as fixations. Fixation velocity algorithms use a duration criterion in combination with a stillness criterion based on eye velocity to determine if a fixation or a smooth pursuit has occurred (Holmqvist et al., 2011).

Both matches were recorded with a Panasonic AG-UX90 4K Camcorder, stationed approximately 5 m above the ground right outside the touchline by the halfway line. The camcorder was used to triangulate the data with the eye tracker, specifically to measure distances between (a) the players and the ball and (b) the players and the nearest opponent.

### Measures and Variables

In this study, we only analyzed fixations where the ball was in play. The only exception to this was fixations conducted within the 2 s prior to a set-piece being taken. This exception was included because it seemed likely that footballers also gather information in the few seconds leading up to the restarting of play.

Although fixations rarely last less than 100 ms (Salvucci and Goldberg, 2000), analyzing fixations with durations as low as 50 ms has been proposed as a possibility in mobile eye-tracking research (Holmqvist et al., 2011). For this study, however, in order to compare our results to other studies, we used a 120 ms minimum fixation threshold for inclusion (Williams and Davids, 1998; Roca et al., 2011, 2013).

Following the data collection, the eye-tracking videos from all the players were synced with the video from the overview camera using the program Sony Vegas Pro 13. We used the first visible ball reception contact to sync the videos. This resulted in a synchronized split-screen video comprising of an eye-tracking video (left) and an overview video (right). We then used the program Tobii Pro Lab to analyze all fixations. The program detected a total of 6,421 fixations. The data set was then reduced by removing fixations with durations of less than 120 ms (*n* = 3,388) and fixations that could not be classified as belonging to either the attack or defense phase (*n* = 201). Hence, 2,832 fixations were included in the final analysis.

Two measures of fixation properties were analyzed: fixation duration and percentage of viewing time. The former refers to the duration of a fixation in milliseconds, as measured by the Tobii Pro Lab fixation filter; the latter refers to the total viewing time spent fixating upon the different fixation locations (Vater et al., 2016). Furthermore, four measures of fixation context were used for the analysis: areas of interest, fixation location, playing phase, and player-to-ball distance.

**Figure 2:**
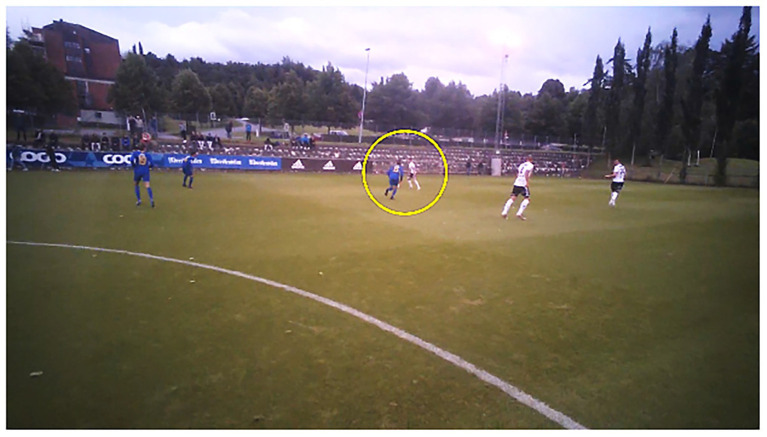
Picture of a gaze-overlaid video from Tobii Pro Lab. Printed with permission.

Based on results from our pilot tests and previous research (e.g., Roca et al., 2013; Vater et al., 2016), four different areas of interest were identified: ball, opponent, teammate, and space. Areas of interest were defined as the exact object(s) of a fixation inside the gaze circle (set to 100% size in the Tobii Pro Lab), which were registered and coded (see Figure 2). For inclusion, the objects (i.e., ball, opponent, and teammate) had to be visible inside the circle. A fixation could, therefore, contain one teammate and one opponent. Furthermore, space was, to some degree, incorporated into most fixations, although fixations were only categorized as space when they were objectless (meaning when neither the ball, a teammate, or an opponent were visible inside the gaze circle, but parts of the pitch were). Fixations that did not fit any category were classified as other.

To further distinguish between what the players were fixating on, combinations of the four areas of interest were categorized, resulting in the following eight possible “fixation location” categories: ball, opponent, and teammate (B/O/T); ball and teammate (B/T); ball and opponent (B/O); ball (B); opponent and teammate (O/T); teammate (T); opponent (O); and space (S). This is a somewhat similar categorization to those of previous *in situ* designs (e.g., van Maarseveen et al., 2017) but differs from other previous laboratory studies (e.g., Roca et al., 2011). The reason for this difference is that, in contrast to laboratory studies where participants are asked to stand relatively close to a screen (for example, 2.8 m, Vater et al., 2016; 2.5 m, Roca et al., 2011, 2013; or 3 m, Hüttermann et al., 2019; away) and, therefore, aim all their fixations at the same exact screen regardless of distance, a real-world design means that all fixations are performed at pitch-level and at different distances. This results in fixations that frequently include more than one object. For example, if a player was looking at the PiP 20 m away, he might fixate on both the player’s foot and the ball simultaneously. Similarly, if the player was looking at an opponent 10 m away, but there was a teammate right behind that player, situated 15 m away, both the opponent and the teammate would be part of the player’s objects of fixation.

The measures of fixation context (i.e., areas of interest and fixation location) were conducted in two different playing phases: the attack phase and defense phase. The attack phase was operationally defined as extending from the moment the investigated player’s team gained possession of the ball (by touching it) to the moment the ball went out of play, a free-kick was awarded, or possession was otherwise lost. When measuring fixation location in the attack phase, fixation on the PiP was considered equivalent to fixations that contained both teammate and ball. The defense phase was operationally defined as extending from the moment the opposing team gained possession of the ball (by touching it) to the moment where the ball went out of play, a free-kick was awarded, or possession was lost. When measuring fixation location in the defense phase, fixation on the PiP was considered equivalent to fixations that contained both opponent and ball. Hence, when referring to the B/T category in attack and B/O category in defense, PiP will be used.

Finally, we also distinguished fixations based on the player-to-ball distance. The player-to-ball distance was operationalized as the number of meters between the investigated player and the ball when a fixation was taking place. This variable was manually coded by the first author, who used the exact pitch markings and video from both the overview camera and the eye-tracker camera to ensure maximum precision. In order to compare the dependent variables under different conditions, a dummy variable was made based on the distance (meters) between the player and the ball. Based on the procedures used by Roca et al. (2013) and Vater et al. (2016) in which participants were situated approximately in the middle of their own half, and where every fixation conducted on the same half was considered to be in the near condition, we operationalized the near condition to be 0–24 m and the far condition to be 25–58 m.

In order to ensure reliable measures, we conducted both intra-reliability and inter-reliability tests for the near and far player-to-ball distance classifications as well as the areas of interest, on 142 (5% of the total) randomly selected situations. For the inter-reliability test, we used an experienced coder who had recently completed a Master’s thesis on visual perception in football (and was a semi-professional football player at the time). The Kappa values of agreement for the player-to-ball distance were *k* = 0.842 (*p* < 0.001) for intra-reliability and *k* = 0.881 (*p* < 0.001) for inter-reliability, which is considered almost perfect agreement (Field, 2014). The intraclass correlation coefficient (ICC) for the areas of interest was 0.981 (*p* < 0.001) for intra-reliability and 0.987 (*p* < 0.001) for inter-reliability, again showing almost perfect agreement (Field, 2014).

### Statistical Analysis

Statistical analyses were performed using SPSS 25.0 (SPSS Inc., Chicago, IL, United States). Differences between areas of interest, fixation location, and the distance condition on the percentage of viewing time and fixation duration for the defense and attack phase were analyzed using univariate analyses of variance (ANOVAs). Mean fixation duration of the eight fixation locations were determined for each participant. Bonferroni’s corrections were used for comparisons of more than two groups, and Cohen’s *d* was calculated as the effect-size measure. The alpha level for all statistical tests was set *a priori* at *α* = 0.05.

## Results

### Fixation Duration

The combined average fixation duration of all 2,832 fixations was 242.29 ms (*SD* = 195.03, *Min.* = 120 ms, *Max.* = 2,400 ms). In the attack phase, the average fixation duration was 247.07 ms (*SD* = 199.54, *n* = 1,486), whereas, in the defense phase, the average fixation duration was 237.02 ms (*SD* = 189.86, *n* = 1,346). A one-way ANOVA of playing phase (2) showed no significant effect, *F*(1,2830) = 1.89, *p* = 0.171, *d* = 0.06.

We also examined the average fixation duration for fixations conducted at different player-to-ball distances (*n* = 2,770, 62 missing). In the near condition (0–24 m), players had an average fixation duration of 228.55 ms (SD = 153.99, *n* = 1,853), whereas, in the far condition (25–58 m), players had an average fixation duration of 266.63 ms (*SD* = 249.54, *n* = 917). A one-way ANOVA of playing phase (2) revealed a significant effect, *F*(1,2830) = 26.89, *p* < 0.001, meaning that the players’ fixation duration was longer in the far condition. However, the effect size of this result was very small, *d* = 0.19.

### Number of Areas of Interest

**Figure 3:**
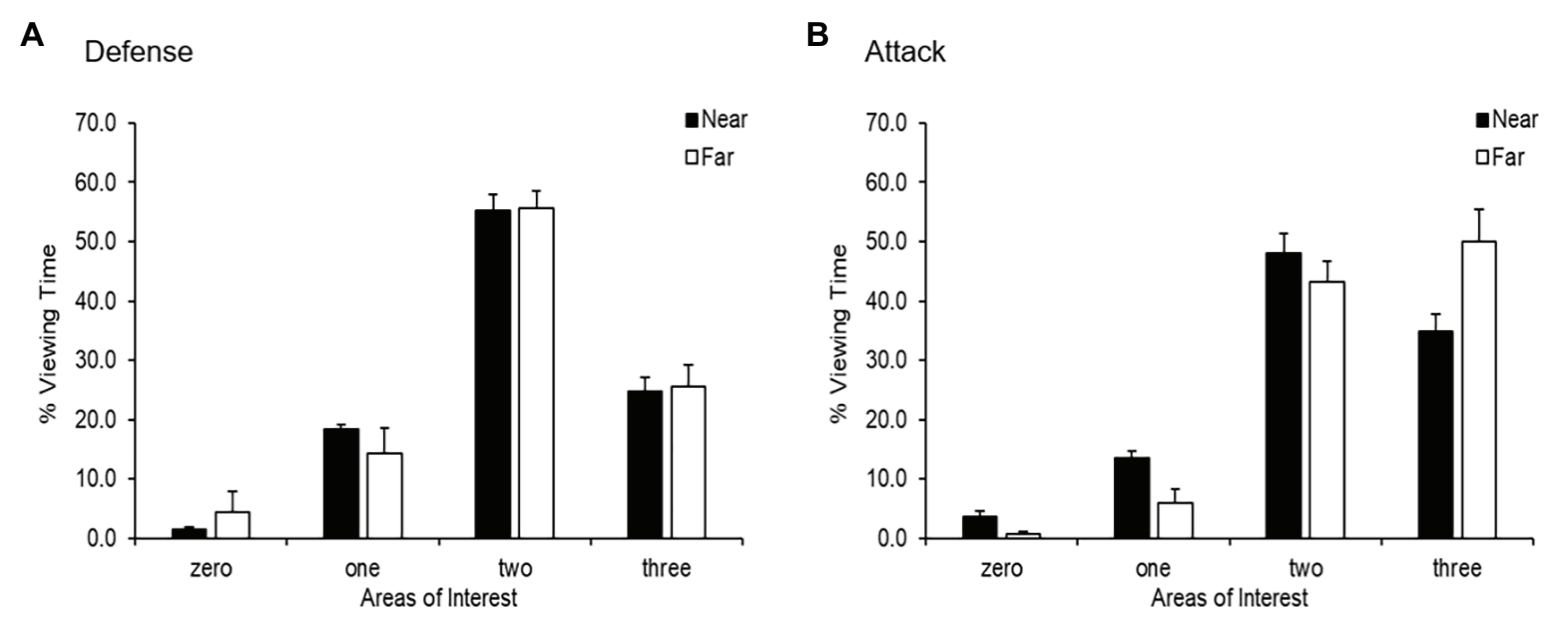
Percentage of viewing time (*M* and *SE*) in defense **(A)** and attack **(B)** as a function of areas of interest (zero, one, two, and three) and distance (near and far).

The percentage of viewing time and the mean fixation duration for each of the informative areas of interest – featuring zero (open space), one, two, or three areas of interest (i.e., teammate, opponent, and ball) – were determined (see Figure 3 for the percentage of viewing time). For the percentage of viewing time, the three-way ANOVA on areas of interest (4) × distance (2) × playing phase (2) with repeated measures on the last two factors revealed a significant three-way interaction [*F*(3,16) = 5.65, *p* = 0.008, *d* = 2.06], meaning that the interactions of the first two ANOVA factors differed across the playing phases. Consequently, two-way ANOVAs on areas of interest (4) × distance (2) with repeated measures on the last factor were conducted separately for each playing phase. For defense, the respective ANOVA revealed a significant effect for areas of interest [*F*(3,16) = 134.53, *p* < 0.001, *d* = 10.06] but not for distance [*F*(1,16) = 0.00, *p* = 0.999, *d* = 0.00] or the two-way interaction [*F*(3,16) = 0.48, *p* = 0.698, *d* = 0.60]. Pairwise comparisons with Bonferroni-corrected values of *p* showed significant differences for all comparisons (*p*s < 0.032), meaning that participants spent most of the time viewing two areas of interest, followed by three areas and one area of interest. Zero areas of interest (space) were very rarely fixated.

Contrarily, the two-way ANOVA on areas of interest (4) × distance (2) with repeated measures on the last factor for attack showed a significant effect for areas of interest [*F*(3,16) = 130.94, *p* < 0.001, *d* = 9.93] as well as an interaction effect [*F*(3,16) = 5.25, *p* = 0.010, *d* = 1.98], but no effect was discernible for distance [*F*(1,16) = 0.00, *p* = 0.999, *d* = 0.00]. Consequently, two separate one-way ANOVAs on areas of interest (4) were conducted for the near and far conditions. The analyses showed a significant effect for both the near condition [*F*(3,16) = 72.58, *p* < 0.001, *d* = 7.40] and the far condition [*F*(3,16) = 53.47, *p* < 0.001, *d* = 6.32], but no differences were observed for the Bonferroni-corrected *post-hoc* analyses.

In the near condition, significant effects were found for all comparisons (*p*s < 0.008) except for the difference between zero areas and one area of interest (*p* = 0.058). This result means that the participants spent the most time viewing two areas of interest followed by three areas of interest and, finally, one area of interest. The analysis of the far condition showed similar differences; however, no difference could be found between zero areas and one area of interest (*p* = 0.999), and the comparison of two and three areas of interest revealed no effect (*p* = 0.999). These findings imply that participants fixated more often on two or three areas than zero areas or one area of interest.

Table 1 reports the means and standard deviations for the fixation duration separated by distance and playing phase. The three-way ANOVA on areas of interest (4) × distance (2) × playing phase (2) with repeated measures on the last two factors revealed neither a three-way nor a two-way interaction [*F*s(3,13) < 2.98, *p*s = 0.071, *d*s = 1.66] as well as no effects for playing phase [*F*(1,13) = 0.14, *p* = 0.713, *d* = 0.21] and distance [*F*(1,13) = 1.65, *p* = 0.221, *d* = 0.71]. However, a significant effect was observed for areas of interest [*F*(3,13) = 8.56, *p* = 0.002, *d* = 2.81]. The Bonferroni-corrected pairwise comparisons demonstrated that the participants showed longer fixation durations for two and three areas of interest than zero areas of interest (*p* = 0.007 and *p* = 0.003, respectively). All significant findings for areas of interest revealed a large effect size, implying important differences.

**Table 1:** Fixation duration (ms) on different areas of interest (zero, one, two, and three) as a function of distance (near and far) and playing phase (defense and attack).

	Near condition		Far condition		
	Defense	Attack	Defense	Attack	Overall
**Areas of interest**	**M (SD)**	**M (SD)**	**M (SD)**	**M (SD)**	**M (SD)**
Zero	144.47 (12.10)	165.00 (28.43)	149.00 (32.42)	135.67 (9.81)	150.02 (23.80)^*^
One	201.01 (31.41)	190.80 (21.61)	216.50 (15.76)	182.00 (56.97)	197.39 (33.42)
Two	227.22 (26.25)	238.40 (27.75)	266.40 (51.37)	242.53 (52.39)	243.64 (40.69)^*^
Three	222.13 (23.18)	246.00 (26.60)	259.60 (110.75)	291.92 (22.99)	254.91 (60.21)^*^
Overall	198.71 (40.38)	210.05 (41.99)	227.33 (77.22)	223.96 (69.94)	214.33 (58.59)
	204.38 (41.06)		225.69 (72.71)		

Significant differences (*p* < 0.05) are marked with ^*^.

### Fixation Location

**Figure 4:**
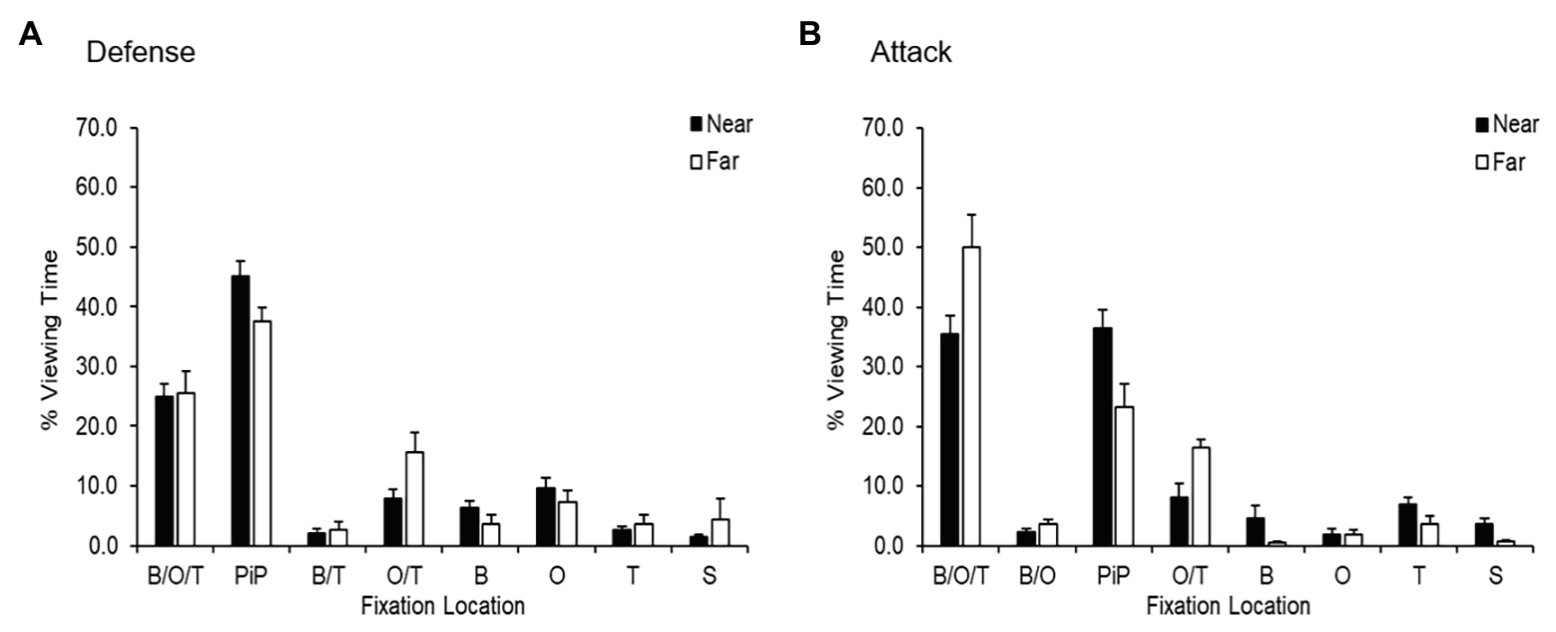
Percentage of viewing time (*M* and *SE*) in defense **(A)** and attack **(B)** as a function of fixation location and distance (near and far). B/O/T, ball, opponent, and teammate; PiP, ball and opponent or ball and teammate; B/T, ball and teammate; B, ball; O/T, opponent and teammate; T, teammate; O, opponent; S, space.

To examine gaze behavior, two three-way ANOVAs on fixation location (8) × distance (2) × distance (2) × playing phase (2) with repeated measures on the last two factors were conducted for the percentage of viewing time and fixation duration. The analysis of the percentage of viewing time revealed a significant three-way interaction [*F*(7,32) = 2.66, *p* = 0.027, *d* = 1.53], meaning that the interactions of the first two ANOVA factors differ across the playing phases. Consequently, two-way ANOVAs on fixation location (8) × distance (2) with repeated measures on the last factor were conducted separately for each playing phase. For both playing phases, the respective ANOVAs revealed significant effects for areas of interest [defensive phase: *F*(7,32) = 81.86, *p* < 0.001, *d* = 8.45; attacking phase: *F*(7,32) = 114.56, *p* < 0.001, *d* = 10.06], as well as three-way interactions [defensive phase: *F*(7,32) = 2.36, *p* < 0.046, *d* = 1.44; attacking phase: *F*(7,32) = 6.42, *p* < 0.001, *d* = 2.37]. However, no effects were noted for distance [defensive phase: *F*(1,32) = 0.00, *p* = 0.995, *d* = 0.00; attacking phase: *F*(1,32) = 0.00, *p* = 0.999, *d* = 0.00]. As depicted in Figure 4, participants spent most of their time viewing the PiP category followed by its B/O/T counterpart. This finding was independent of distance. However, the significant two-way interactions imply that the participants’ gaze behaviors were different in the near and far conditions. Therefore, two separate one-way ANOVAs on fixation location (8) were conducted for each playing phase. For the defensive phase, the analysis showed a significant effect for both the near condition [*F*(1,32) = 97.36, *p* < 0.001, *d* = 9.21] and the far condition [*F*(1,32) = 25.01, *p* < 0.001, *d* = 4.67].

In the near condition, the Bonferroni-corrected pairwise comparisons demonstrated significant differences between the B/O/T category and the PiP category (*p* < 0.001), as well as any other fixation location (*p*s < 0.001). Additionally, the participants spent more time viewing the opponent than the open space (*p* = 0.023). The findings for the far condition were similar. The analysis revealed that the participants fixated more on the B/O/T and PiP categories than any other fixation location (*p*s < 0.001), the results for the comparison between the B/O/T and the O/T categories being the exception (*p* = 0.292).

Besides these effects, one additional difference was found: the participants spent more time focusing on the O/T than the B/T category (*p* = 0.031). The effect sizes for all significant effects remained large, meaning that all the findings should be classified as important.

Similar to the defensive phase, the two separate one-way ANOVAs on fixation location (8) for attack showed significant effects for the near condition [*F*(4,20) = 63.88, *p* < 0.000, *d* = 7.46] and the far condition [*F*(4,20) = 47.43, *p* < 0.000, *d* = 6.44].

The Bonferroni-corrected *post-hoc* analysis for the near condition demonstrated significant differences between the B, O/T, and PiP categories and any other fixation location (*p*s < 0.001). Similarly, in the far condition, the Bonferroni-corrected pairwise comparisons showed that the participants spent more time viewing the B, O/T, and PiP categories than any other fixation location (*p*s < 0.001). However, no difference was found between the PiP and O/T categories (*p* = 0.999). Moreover, comparison between the B, O/T, and PiP categories also revealed an effect (*p* < 0.001). Additionally, the participants fixated more often on the O/T category than the B, O, B/O, T, or S categories (*p*s < 0.029). Similar to the defense phase, all significant findings showed a large effect size. Thus, these findings appear to be important.

Contrary to the analysis of the percentage of viewing time, the three-way ANOVA on fixation location (8) × distance (2) × playing phase (2) on fixation duration with repeated measures on the last two factors showed no three-way interaction [*F*(7,23) = 0.73, *p* = 0.651, *d* = 0.94]. However, a significant effect was found for distance [*F*(1,23) = 4.60, *p* = 0.043, *d* = 0.90], meaning that the participants exhibited longer fixation durations in the far condition than in the near condition. Additionally, the analysis revealed significant differences in fixation duration on fixation location [*F*(7,23) = 3.76, *p* = 0.007, *d* = 2.14] (see Table 2).

**Table 2:** Fixation duration (ms) on different fixation locations as a function of distance (near and far) and playing phase (defense and attack).

	Near condition		Far condition		
	Defense	Attack	Defense	Attack	Overall
**Fixation location**	**M (SD)**	**M (SD)**	**M (SD)**	**M (SD)**	**M (SD)**
B/O/T	222.28 (23.08)	246.09 (26.58)	259.69 (110.82)	291.89 (23.09)	254.99 (60.21)^*^
PiP	233.53 (29.05)	254.82 (29.25)	281.82 (49.15)	246.14 (64.45)	254.08 (45.51)^*^
B/O; B/T	225.63 (58.54)	189.26 (17.19)	263.89 (86.76)	178.22 (39.49)	210.86 (59.44)
O/T	197.40 (35.25)	209.15 (36.01)	239.84 (87.15)	268.15 (59.91)	228.63 (60.74)
B	206.12 (43.52)	185.16 (16.91)	217.90 (70.77)	226.67 (150.11)	206.35 (67.47)
O	197.31 (27.25)	180.55 (51.14)	204.36 (21.92)	233.49 (101.95)	200.42 (50.64)
T	180.67 (49.01)	199.55 (26.31)	244.90 (46.44)	173.16 (40.92)	198.52 (46.43)
S	144.46 (12.11)	164.98 (28.40)	149.17 (32.59)	135.56 (9.62)	150.03 (23.83)^*^
Overall	200.29 (42.85)	203.69 (40.96)	235.07 (74.78)	224.29 (77.10)	214.89 (61.16)
	202.01 (41.67)^**^		229.84 (75.54)^**^		

Significant differences (*p* < 0.05) are marked with ^*^ and ^**^. B/O/T, ball, opponent, and teammate; PiP, ball and opponent (defense) and ball and teammate (attack); B/O, ball and opponent (attack); B/T, ball and teammate (defense); O/T, opponent and teammate; B, ball; O, opponent; T, teammate; S, space.

The analysis of the fixation duration revealed a significant effect for fixation location once again [*F*(4,40) = 3.46, *p* = 0.004, *d* = 1.29], but no interaction effect was observed [*F*(4,40) = 0.34, *p* = 0.933, *d* = 0.40]. Compared to the percentage of viewing time, a significant effect was found for distance [*F*(1,40) = 5.97, *p* = 0.018, *d* = 0.64], meaning that the participants showed longer fixation duration in the far condition than the near conduction (see Table 2).

The Bonferroni-corrected pairwise comparisons demonstrated three significant differences. The participants fixated on the B/O/T, PiP, and O/T categories longer than they did on space (S; *p* < 0.001*, p* = 0.003, and *p* = 0.014, respectively). Compared to the large effect size of the significant difference for fixation location, the finding for distance revealed only a medium effect size.

## Discussion

This study was conducted to learn more about the gaze behaviors of elite football players in a real-world performance setting. We analyzed a total of 2,832 fixations from five players during two training games and focused our analysis on the duration and location of fixations during 11 v 11 match play.

The most striking result from our analysis is that these elite footballers used longer fixation durations when more areas of interest (i.e., ball, teammate, and opponent) were visible in their foveal vision (fixation circle). More specifically, the players performed significantly longer fixations when there were two or three areas of interest compared to zero areas in the attack phase and compared to both one and zero areas in the defense phase. These results run contrary to those of Helsen and Starkes (1999), who reported that players reduce the duration of their fixations when more display information becomes available. However, other studies of gaze behavior have shown that fixation duration increases with more information (Just and Carpenter, 1976).

Our findings suggest that the more complex the situation (i.e., being positioned between opponents’ lines of defense), the more time the player may need to obtain sufficient information before executing his decision. Interestingly, these results were similar regardless of whether the ball was near (0–24 m) or far from the players (25+ m). Hence, the number of areas of interest seemed to have a larger impact than player-to-ball distance on fixation duration in real football match play.

The observed association between fixation duration and areas of interest of the central midfielders in this study should be viewed in light of their positional demands. Research has shown that central midfielders are the priority link in attack play in football (Clemente et al., 2015). Central midfielders have been shown to have the highest number of passes and pass accuracy of any playing position (Bradley et al., 2013). Consequently, players in that position are used to expecting the ball in different areas and phases of play and have, therefore, learned to look for opportunities for action in ways specific to their playing position.

The present study also found that the average duration of fixations was significantly shorter than expected based on prior studies conducted in a laboratory setting. Our results revealed that players had an average fixation duration of 242.29 ms. Different laboratory studies on elite or skilled footballers, deploying similar fixation thresholds, have reported average fixation durations ranging from 467 to 1,002 ms (Helsen and Starkes, 1999), 423 to 492 ms (Mann et al., 2009), 369 ms (Roca et al., 2011), and 332 to 598 ms (Roca et al., 2013). These discrepancies raise the question of whether examining football players’ visual fixations in a laboratory setting is inadequate when attempting to capture footballers’ gaze fixations during the dynamics of match play, where a different landscape of information and sensations influence both decision-making and gaze behavior (Hüttermann et al., 2018).

The same differences in duration were also evident when comparing the mean fixation duration from our study to *in situ* experiments in other sports, such as basketball (342–677 ms; van Maarseveen et al., 2017) and ice hockey (346.74 ms for elite and 591.59 ms for non-elite; Martell and Vickers, 2004). A possible explanation for this might be that the experimental tasks and study context focused on different, specific game situations of each sport: 2 v 2 (Martell and Vickers, 2004) and 3 v 3 (van Maarseveen et al., 2017). The time and spatial constraints may vary depending on game situations and sports, which may limit gaze behavior to fewer potential fixation locations than in our study.

Another possible explanation for the shorter fixation durations found in our study could be the high skill level of the participants. The participants were elite players, playing at the highest national level. Similarly, both Williams et al. (1994) and Cañal-Bruland et al. (2011) found that experienced football players used shorter fixations than inexperienced players, which could be attributed to the quicker and more precise information extracting ability of elite players (Cañal-Bruland et al., 2011). Following this argument, it is possible that a comparison of lower-level and elite players in more representative settings would provide similar results because lower-level players may need more time to draw information from each fixation compared to experts (Williams et al., 1994).

Another important finding was the relationship between areas of interest and the percentage of viewing time. As seen in Figures 3A,B, a reverse-U shape appears in the defense phase as well as in the near-condition attack phase. Conversely, the results show a progressive increase in percentage viewing time in the far condition attack phase. This raises the question of whether the player-to-ball distance has a bigger influence on gaze behavior in the attack phase than in the defense phase. This result may be explained by the fact that when the ball is far from the players in the attack phase, they direct their attention to sources of information other than the PiP in the search for space to exploit for themselves or their teammates, thus fixating on more areas of interest. However, when the ball comes closer and the opportunity to receive a pass increases, they direct their attention to the PiP.

In the current study, an examination of the players’ viewing time of fixation locations in the defense phase revealed that they focused their visual attention on the PiP category significantly more than any other category. This effect was prevalent in both the near and far conditions. This result is even more sizable than reported since the B/O/T category often includes the PiP category as well. This finding is similar to the results reported by Roca et al. (2013) and Vater et al. (2016), who found that players fixated significantly more on the PiP than any other fixation locations in the defense phase.

Interestingly, analysis of players’ viewing time in the far-condition attack phase revealed that the participants spent 49.99% of the time fixating on the B/O/T category. This was significantly more than any other fixation location in the far condition. This result may be explained in part by the long distance (25 m+), which makes it more likely that additional objects will appear in the line of foveal vision between the ball and the analyzed player. However, the same effect did not occur in the defense phase. The B/O/T category is a new fixation location category, constructed especially for our natural environment study context; therefore, more research is needed to understand why players fixate foveally on this category to such a degree when the ball is far away in the attack phase.

Finally, the fixation time given to the O/T category in attack was shown to be significantly higher in the far condition compared to the near condition. Although not significant, the same tendency was found in the defense phase. It is difficult to suggest a tentative interpretation of this result since this is the first study to utilize an O/T fixation location category. However, a plausible explanation may be that when the ball is further away, players have more time to look at more informative areas away from the ball in order to detect important information that may guide future defensive and attacking behavior. This activity has previously been reported as visual exploratory behavior (McGuckian et al., 2018a).

Previous research has shown that elite midfielders have an exploratory frequency of up to 0.62 per second in the 10 s leading up to receiving the ball (Jordet et al., 2013). It is, therefore, reasonable to believe that the elite midfielders in this study also performed extensive visual exploratory behaviors in the attack phase, especially when the ball was far away. To investigate this further, studies that combine measures of gaze and visual exploratory behavior are needed.

Our findings suggest some practical implications for coaches and athletes. For example, we found that the average duration of a fixation in real-world football is quite short (242.29 ms), suggesting that numerous quick fixations are relevant to seizing opportunities for action provided in the game environment. Additionally, our results suggest that increasing the number of informative areas in the display, from only searching for space (S) to looking for the ball, opponents, and teammates (B/O/T) simultaneously, increases the time needed to draw information from those sources. Thus, exercises should provide players with the ability to locate many sources of information under severe time constraints, inducing the same dynamics prevalent in the players’ use of their visual perceptual systems representative of real-world match play. For example, there is less need for longer fixations in a 2 v 2 situation than an 8 v 8 situation because there are fewer potential areas of interest present. Closed drills where movement solutions are pre-determined, conducted in an environment that is non-representative of the match-play context, might alter the visual fixation and search strategies football players use in 11 v 11 match play. In sum, coaches need to be aware of how visual fixation and search strategies change depending on the numerical, spatial, and temporal conditions of an exercise.

## Limitations

The findings of this study should be considered in light of some limitations. First, the study was explorative and observational, preventing us from addressing any causal relationships and restricting the generalizability of our results. Our implications for practice should, therefore, be considered tentative and speculative and may be contested by future experimental research. Second, the lack of a clear theoretical framework limited our ability to generate and test clear hypotheses. Third, we chose not to include measures of decision-making and performance in this study, instead focusing solely on players’ gaze behaviors. Fourth, the manner in which the dynamics of the game influenced gaze behavior, for example, if a team scored early on, was not controlled for. Having a lead would potentially direct the gaze toward more defensively important aspects of the game, thus influencing our results. Fifth, our *in situ* design did not allow us to include any measure of fixation frequency. Because of the study context, where all players experienced completely different playing situations, played a different number of seconds in the attack and defense phase, and had a different number of gaze samples, the inclusion of any measure of fixation frequency would not constitute a valid approach. Finally, inaccuracies in the technological equipment’s detection may have occurred due to the limited use of head-mounted eye-tracking devices in real-world football matches prior to our study.

## Future Research

Based on the limitations and results of this study, we propose several recommendations for future research. First, future research should address how performance is associated with gaze behavior in football, such as passing accuracy (Eldridge et al., 2013) or defensive actions (Nagano et al., 2004). Second, future research across all invasion sports should replicate our study design in order to investigate differences in gaze behavior between players at different skill levels. Third, future research should explore methods of simultaneously examining foveal and peripheral vision. Fourth, future studies should examine different playing positions and strategies because there is reason to believe that players in positions other than central midfielders utilize different gaze behaviors (McGuckian et al., 2020). Fifth, studies should strive to combine measures of gaze and visual exploratory behavior. This is because it is reasonable to believe that the elite midfielders in this study also performed extensive visual exploratory behaviors in the attack phase, especially when the ball was far away, similar to the exploratory frequencies reported by Jordet et al. (2013). Sixth, future research could benefit from positioning itself within a clear theoretical perspective in order to generate and test hypotheses relevant to promoting an understanding of how visual perception underpins sports performance.

## Conclusion

In recent years, the association between gaze behaviors and performance has received extensive interest from researchers and practitioners. With the use of new technologies, we now have the opportunity to investigate the gaze behaviors of football players during match play. Our exploratory case study reported differences in both the areas of interest and fixation locations when the ball is near or far, as well as when playing in the attack or defense phase. The average fixation duration was lower than previously reported in laboratory-based research designs, as well as *in situ* designs in other sports. Furthermore, the results revealed that elite central midfield players have a longer fixation duration when more areas of interest are available to them.

## Data Availability Statement

The data generated for this study are available upon request to the first author.

## Ethics Statement

The studies involving human participants were reviewed and approved by the Norwegian Centre for Research Data. The participants provided their written informed consent to participate in the study.

## Author Contributions

KMA contributed to the conceptualization, data collection, data analysis, and writing of the paper. LM contributed to the data analysis and writing of the paper. CTB contributed to parts of the data analysis and writing of the paper. GJ contributed to the conceptualization, data collection, and writing of the paper. All authors contributed to the article and approved the submitted version.

### Conflict of Interest

The authors declare that the research was conducted in the absence of any personal or financial relationships that could potentially be depicted as a conflict of interest.

## Footnotes

^1^Since the data collection, all players in this study have established themselves in the squad of a Norwegian premier league team and have played between 33 and 137 matches (*M* = 86.2, *SD* = 42.16) at the top national level to date.
